# Vitamin D–vitamin D receptor system down-regulates expression of uncoupling proteins in brown adipocyte through interaction with Hairless protein

**DOI:** 10.1042/BSR20194294

**Published:** 2020-06-10

**Authors:** Pei-qi Wang, Dao-xiang Pan, Chun-qiu Hu, Yu-lin Zhu, Xiao-jing Liu

**Affiliations:** 1 Department of Pediatrics, First Affiliated Hospital, Anhui Medical University, 218 Jixi Road, Hefei 230 022, Anhui Province, China; 2 Department of Toxicology, Anhui Medical University, 81 Meishan Road, Hefei 230 032, Anhui Province, China

**Keywords:** 1,25(OH)2D3, hairless protein, uncoupling proteins, vitamin D receptor, vitamin D

## Abstract

Our previous study showed that feeding mice with vitamin D deficiency diet markedly alleviated high-fat-diet-induced overweight, hyperinsulinemia, and hepatic lipid accumulation. Moreover, vitamin D deficiency up-regulated the expression of uncoupling protein 3 (Ucp3) in white adipose tissue (WAT) and brown adipose tissue (BAT). The present study aimed to further investigate the effects of vitamin D and vitamin D receptor (Vdr) on Ucp1–3 (Ucps) expression in brown adipocyte and the mechanism involved in it. Rat primary brown adipocytes were separated and purified. The effects of the 1,25(OH)_2_D_3_ (1,25-dihydroxyvitamin D3; the hormonal form of vitamin D) and Vdr system on Ucps expression in brown adipocytes were investigated in basal condition and activated condition by isoproterenol (ISO) and triiodothyronine (T3). Ucps expression levels were significantly down-regulated by 1,25(OH)_2_D_3_ in the activated brown adipocyte. Vdr silencing reversed the down-regulation of Ucps by 1,25(OH)_2_D_3_, whereas Vdr overexpression strengthened the down-regulation effects. Hairless protein did express in brown adipocyte and was localized in cell nuclei. 1,25(OH)_2_D_3_ increased Hairless protein expression in the cell nuclei. Hairless (Hr) silencing notably elevated Ucps expression in activated condition induced by ISO and T3. Moreover, immunoprecipitation results revealed that Vdr could interact with Hairless, which might contribute to decreasing expression of Vdr target gene Ucps. These data suggest that vitamin D suppresses expression of Ucps in brown adipocyte in a Vdr-dependent manner and the corepressor Hairless protein probably plays a role in the down-regulation.

## Introduction

Vitamin D receptor (Vdr) belongs to the superfamily of nuclear receptors. 1,25-dihydroxyvitamin D3 (1,25(OH)_2_D_3_), the hormonal form of vitamin D, is the natural ligand of Vdr. After binding to 1,25(OH)_2_D_3_, Vdr recruits its partner retinoid X receptor and regulates the transcription of target genes via binding to its genomic binding sites [1]. In addition to the classical function in regulating calcium homeostasis, 1,25(OH)_2_D_3_ and Vdr system are involved in the regulation of many non-calcemic functions, such as the regulation of the immune system, liver, skeletal muscle, tumor cells, and adipocytes [2–4].

Brown adipose tissue (BAT), once thought to fully regress in humans after the neonatal period are now known to be present throughout adult life. BAT functions in non-shivering thermogenesis by uncoupling ATP synthesis from respiration and plays an important role in energy expenditure. This uncoupling function is conferred mainly by uncoupling protein (Ucp) 1 (Ucp1), which is uniquely expressed in the inner membrane of brown adipocyte mitochondria. Ucp2 and Ucp3 are also expressed in BAT, and both proteins have uncoupling capabilities [5,6]. However, the primary roles of Ucp2 and Ucp3 in BAT are not well characterized. Interestingly, both Ucp2 and Ucp3 are up-regulated in the Ucp1 knockout mice, suggesting compensatory roles of these proteins in the regulation of energy homeostasis [7].

Vitamin D and Vdr system also play a vital role in energy balance. Vdr knockout mice have reduced body weight and are resistant to high-fat-diet-induced obesity [8]. White adipose tissue (WAT) from Vdr knockout mice has areas of multilocular cell clusters and an increase in the expression of Ucp1 [9]. Transgenic mice that overexpress Vdr under an aP2 promoter/enhancer element have the opposite phenotype of the Vdr knockout mice, with decreased energy expenditure and expression levels of Ucp1–3 (Ucps) [10]. Our previous study showed that feeding mice with vitamin D deficiency diet markedly alleviated high-fat-diet-induced overweight, hyperinsulinemia, and hepatic lipid accumulation. Moreover, vitamin D deficiency up-regulated the expression of Ucp3 in WAT and BAT [11]. The above-mentioned researches are all *in vivo* experiments. *In vitro*, how does the vitamin D-Vdr system regulate Ucps expression? In differentiated 3T3-L1 adipocytes stably transfected with Vdr shRNA, Ucp1 expression level was significantly elevated. It further supports the hypothesis that Vdr represses Ucp1 expression in white adipocytes [12]. Primary brown adipocyte was isolated from WT and Vdr knockout mice and treated with 1,25(OH)_2_D_3_, the expression of Ucp1 and Ucp3 was markedly decreased in WT brown adipocyte after 24 h of treatment; while 1,25(OH)_2_D_3_ had no effect on the cells from Vdr knockout mice. The result suggests that 1,25(OH)_2_D_3_ appears to directly down-regulate Ucp1 and Ucp3 expression in brown adipocyte and the regulation is mediated by Vdr [13]. However, the specific mechanism involved in the regulation is still unclear.

Obesity and associated metabolic diseases are growing public problems. Increasing the body’s energy expenditure is an attractive strategy to treat these disorders. Therefore, the regulation of Ucps in brown adipocyte and the mechanism involved in it deserve further study. Our results demonstrate once again that vitamin D–Vdr system directly suppresses the expression of Ucps in brown adipocyte *in vitro*. Importantly, we find that the corepressor Hairless protein probably plays a role in the down-regulation.

## Materials and methods

### Chemicals and reagents

1,25(OH)_2_D_3_, isoproterenol (ISO), and triiodothyronine (T3) were purchased from Sigma Chemical Co. (St. Louis, MO). TRIzol reagent, Lipofectamine 2000 transfection reagent, and all primers were from Invitrogen. RT-PCR kit was from Promega. Protein extraction kit and enhanced chemiluminescence (ECL) detection kit were from Pierce Biotechnology (Rockford, IL, U.S.A.). Antibodies against Vdr, Ucps, Hairless, and β-actin were from MDL Biotech Co. (Beijing, China). Reagents involved in cell culture were from Gibco. All the other reagents were from Sigma or as indicated in the specified methods.

### Animals and primary brown adipocyte culture

Wistar Sprague–Dawley male rats 4 weeks old were purchased from Beijing Vital River whose foundation colonies were all introduced from Charles River Laboratories, Inc. All animal experiments were conducted in the Experimental Animal Center of the First Affiliated Hospital, Anhui Medical University. The present study was approved by the Ethics Committee of First Affiliated Hospital, Anhui Medical University, and followed the ethical guidelines of the International Association for the Study of Pain (IASP) for pain research in animals. Rats were deeply anesthetized with urethane (5 ml/kg) intraperitoneally and killed by exsanguination. Interscapular brown fat pads were dissected immediately after animals were killed and placed in cold PBS kept on ice. The fat pads were cut with sterile scissors into small pieces and digested with 0.2% collagenase (Sigma–Aldrich, C6885) for 30 min in a shaking water bath. The fat pieces were cultured in six-well plates at 37°C and 5% CO_2_ in the presence or absence of 10^−7^ M of 1,25(OH)_2_D_3_ for 24 h. For T3 and ISO treatment, cells were treated with 10^−7^ M of T3 for 72 h and 10^−6^ M of ISO for 6 h. Primary brown adipocytes were washed with chilled PBS for three times and then harvested for real-time RT-PCR and Western blot.

### Small interfering RNA transfection

The small interfering RNAs (siRNAs) of control, Vdr, and Hairless (Hr) were synthesized from Thermo Fisher Scientific. The sequence of Vdr siRNA was GCUUCCACUUCAAUGCUAUGATT, the sequence of the Hr siRNA was GACUCUGGAUGGCCCAAGUUUTT. The siRNAs were transfected into primary brown adipocytes by Lipofectamine RNAiMax (Thermo Fisher Scientific) according to the manufacturer’s instructions. The transfection efficiencies of Vdr siRNA and Hr siRNA were assessed 72 h after transfection.

### Overexpression

For the study of gene overexpression, Myc-DDK tagged murine Vdr or Hr expression plasmid (OriGene, Rockville, MD) was transfected into brown adipocytes for 72 h and the stably transfected cells were selected by antibiotics (G418) for 3 weeks. The exogenous Vdr and Hairless expression were confirmed by protein expression.

### Immunoprecipitation

The protein interactions between Vdr and Hairless were detected using Immunoprecipitation Kit Dynabeads® Protein G (Invitrogen, Thermo Fisher Scientific, Inc., Waltham, MA), according to the manufacturer’s instructions as well as using a magnet. Cells were treated with or without 10^−7^ M of 1,25(OH)_2_D_3_ for 24 h. Cells were then scraped into ice-cold PBS and lysed with lysis buffer. Mouse monoclonal anti-Vdr antibody (5 µg) or control mouse IgG antibody was incubated with 50 μl of Dynabeads®Protein G for 10 min at room temperature and added to the lysate. Immune complexes were precipitated 2 h at 4°C. The immunoprecipitated material was magnetically separated by washing and elution, further added with SDS buffer, and subjected to Western blot analysis.

### Real-time RT-PCR

Total RNA from brown adipocytes was extracted using TRIzol reagent. RNase-free DNase-treated total RNA (1.0 µg) was reverse-transcribed with AMV (Promega). Real-time RT-PCR was performed with a LightCycler® 480 SYBR Green I Master qPCR mix (Roche) according to a previous study [11]. Specific primers of all genes were listed in Table 1. All RT-PCR experiments were performed in triplicate.

**Table 1:** Primers for real-time PCR

Genes	Primer Forward	Primer Reverse
*β-actin*	GACAGGATGCAGAAGGAGATTACT	TGATCCACATCTGCTGGAAGGT
*Ucp1*	AGCTTTGCTTCCCTCAGGAT	TGGCTTTGTGCTTGCATTCT
*Ucp2*	CTCCCAATGTTGCCCGAAAT	GAGGTCGTCTGTCATGAGGT
*Ucp3*	CCAGCTTCACCTGCATACAC	GAAGGCTGAAGGCTTAACGG
*Vdr*	GTCATCGGCTTTGCCAAGAT	AGACTGGTTGGAGCGTAACA
*Hairless*	CCCAGAGCATCCATGTGACTG	TGCCCACCAGAGTTTAAGCC

### Western blot

Brown adipocytes were washed three times with ice-cold PBS and lysed by incubating for 30 min on ice with lysis buffer. Total lysate (30 µg per well) was separated electrophoretically by 12.5% SDS/PAGE and transferred to a polyvinylidene fluoride membrane. The membranes were incubated for 2 h with the following antibodies: Vdr (1:1000; #MD5738), Ucp1 (1:1000; #MD6956), Ucp2 (1:1000; #MD6957), Ucp3 (1:1000; #MD5264), Hairless (1:1000; #MD6958). β-actin (1:1000; #MD6338) was used as a loading control. After washing in DPBS containing 0.05% Tween-20 three times for 10 min each, the membranes were incubated with goat anti-rabbit IgG antibody (#MD2141) or goat anti-mouse IgG antibody (#MD2142) for 2 h and then washed again. Finally, signal development was performed using the ECL detection kit.

### Statistical analysis

Normally distributed data were expressed as means ± S.E.M. ANOVA and the Student–Newmann–Keuls post hoc test were used to determine differences among different groups. *P*<0.05 was considered statistically significant.

## Results

### Effect of 1,25(OH)_2_D_3_ on Vdr and Ucps in brown adipocyte

**Figure 1:**
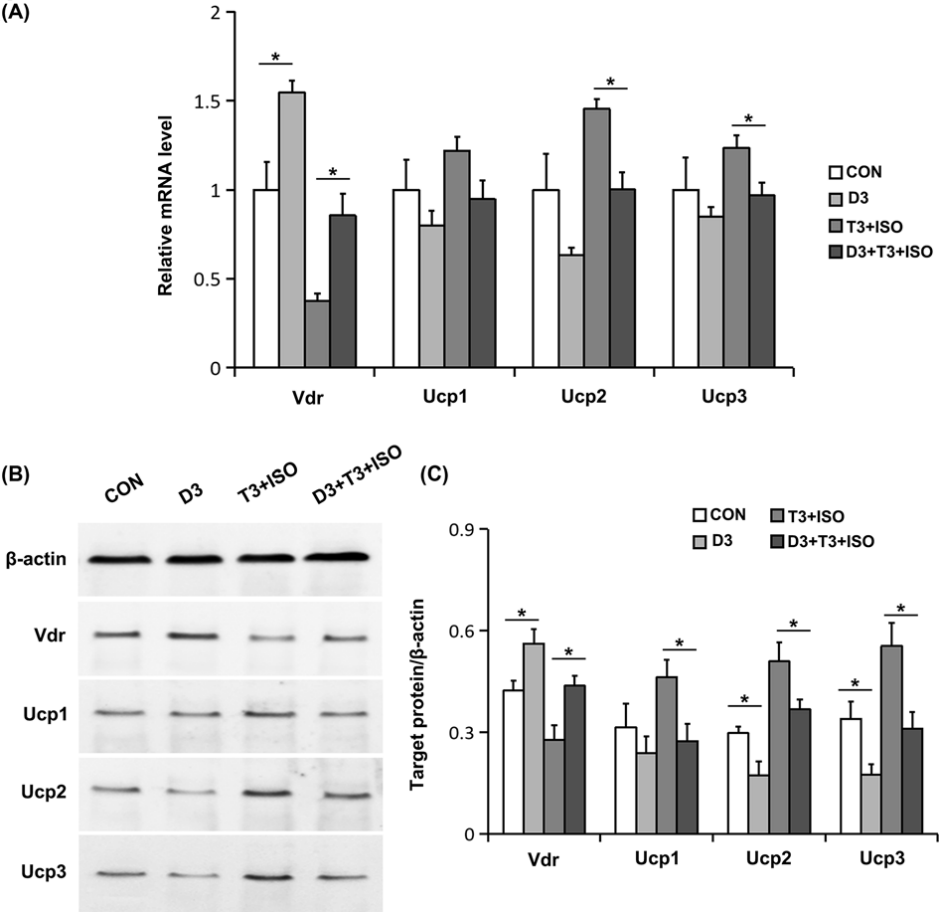
Effects of 1,25(OH)_2_D_3_ on the expression of Vdr and Ucps (**A**) The mRNA levels of Vdr and Ucps were determined using real-time RT-PCR. (**B**) The protein levels of Vdr and Ucps were measured using Western blot. Blots reflect representative data. (**C**) The level of target protein/β-actin was quantified. Data were expressed as means ± SEM (*n*=3). *P<0.05.

Usually, Ucp1 is activated adrenergically and requires T3 [14], so we investigated the effects of vitamin D and Vdr system on Ucps expression in brown adipocytes treated with or without ISO and T3. As expected, Vdr expression both in mRNA level (Figure 1A) and protein level (Figure 1B,C) were significantly up-regulated by 1,25(OH)_2_D_3_. The effects of 1,25(OH)_2_D_3_ on Ucps expression were then analyzed. Although no significant difference in Ucps mRNA expression was observed in unactivated brown adipocyte (Figure 1A), Ucps protein expression, Ucp2 mRNA, and Ucp3 mRNA levels were significantly down-regulated by 1,25(OH)_2_D_3_ in activated brown adipocyte by ISO and T3 (Figure 1).

### Effects of Vdr interference on Ucps expression regulated by 1,25(OH)_2_D_3_

**Figure 2:**
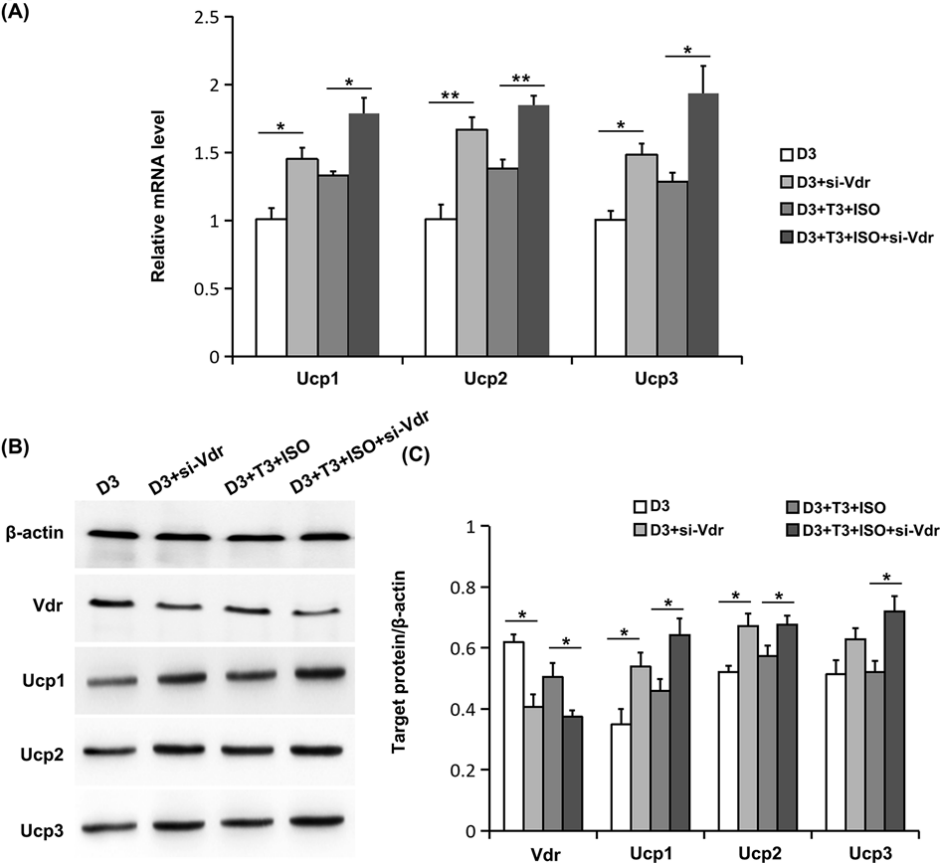
Effects of Vdr interference by siRNA on the expression of Ucps regulated by 1,25(OH)_2_D_3_ (**A**) The mRNA levels of Ucps were determined using real-time RT-PCR. (**B**) The protein levels of Vdr and Ucps were measured using Western blot. Blots reflect representative data. (**C**) The level of target protein/β-actin was quantified. Data were expressed as means ± SEM (*n*=3). *P<0.05, **P<0.01.

As expected, Vdr interference by siRNA decreased expression of Vdr in protein level (Figure 2B,C). Compared with the effect of 1,25(OH)_2_D_3_, Vdr interference by siRNA markedly up-regulated mRNA levels of Ucp1, Ucp2, and Ucp3, whether activated by ISO and T3 or not (Figure 2A). Furthermore, the results of Western blot showed a significant elevation of Ucps protein induced by Vdr siRNA (Figure 2B,C). The above findings indicate that decreased Vdr expression reverses the 1,25(OH)_2_D_3_-mediated inhibitory effects on Ucps expression.

### Effects of Vdr overexpression on Ucps expression regulated by 1,25(OH)_2_D_3_

**Figure 3:**
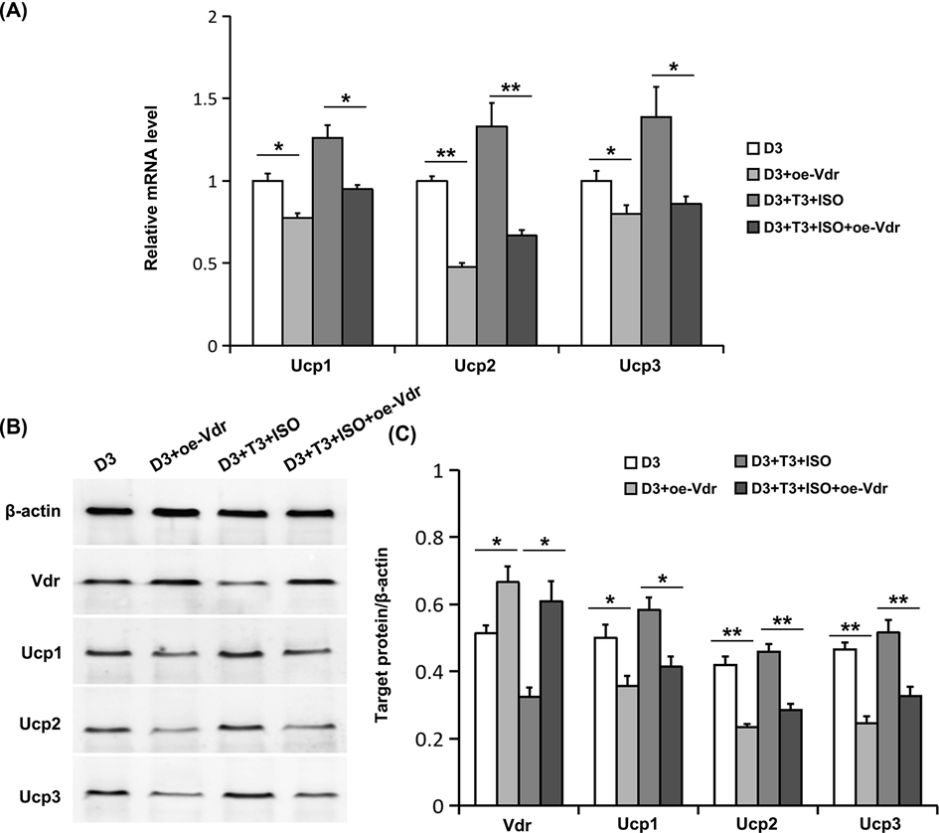
Effects of Vdr overexpression on the expression of Ucps regulated by 1,25(OH)_2_D_3_ (**A**) The mRNA levels of Ucps were determined using real-time RT-PCR. (**B**) The protein levels of Vdr and Ucps were measured using Western blot. Blots reflect representative data. (**C**) The level of target protein/β-actin was quantified. Data were expressed as means ± SEM (*n*=3). *P<0.05, **P<0.01.

To further investigate the role of Vdr on Ucps expression in primary rat brown adipocyte, we observed the effects of Vdr overexpression. As expected, Vdr overexpression elevated the protein level of Vdr (Figure 3B,C). Compared with the D3 group, whether activated by ISO and T3 or not, Ucps mRNA (Figure 3A) and protein levels (Figure 3B,C) were both significantly up-regulated in the Vdr overexpression group. It suggests Vdr overexpression strengthens the down-regulation of Ucps mediated by 1,25(OH)_2_D_3_. The results of Vdr interference by siRNA experiment and Vdr overexpression experiment indicate that vitamin D-regulated Ucps inhibition is dependent on Vdr.

### Role of Hairless protein on Ucps expression regulated by 1,25(OH)_2_D_3_

**Figure 4:**
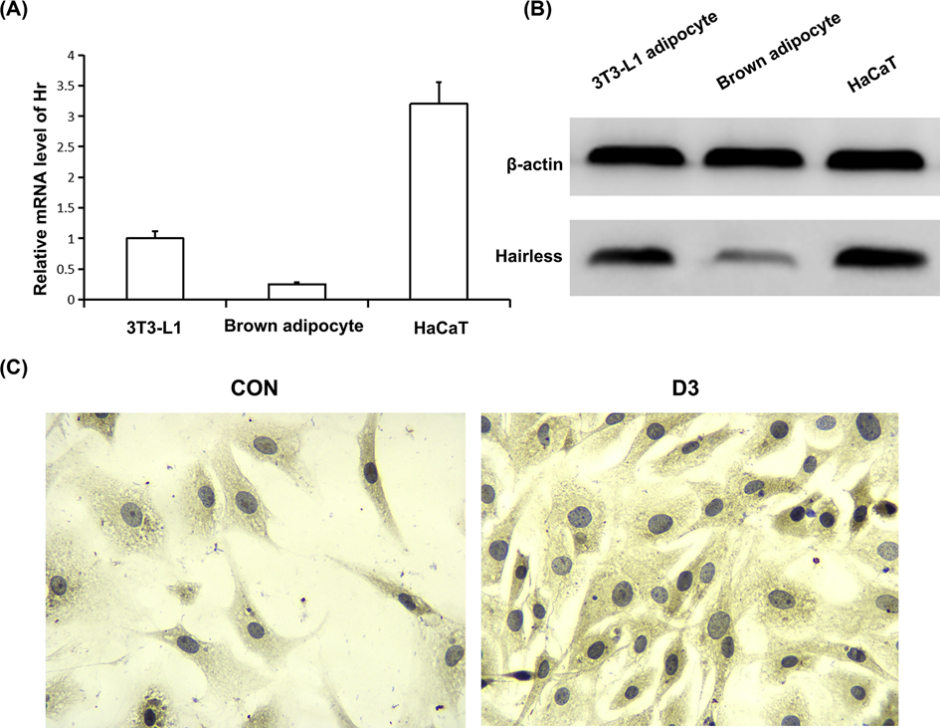
Hairless expression in the primary brown adipocyte of mice (**A**) Hr mRNA level was determined using real-time RT-PCR. (**B**) Hairless protein level was measured using Western blot. (**C**) Immunohistochemical staining of Hairless protein using anti-Hairless antibody.

To determine the role of Hairless protein on Ucps expression regulated by 1,25(OH)_2_D_3_, we first investigated the expression of Hairless protein in rat primary brown adipocyte. We found that Hairless protein did express in brown adipocyte, although in lower level when compared with 3T3-L1 white adipocyte and HaCaT keratinocyte (Figure 4A,B). Immunohistochemical assay results revealed that Hairless protein was located in cell nuclei and 1,25(OH)_2_D_3_ increased Hairless protein level in the cell nuclei (Figure 4C).

**Figure 5:**
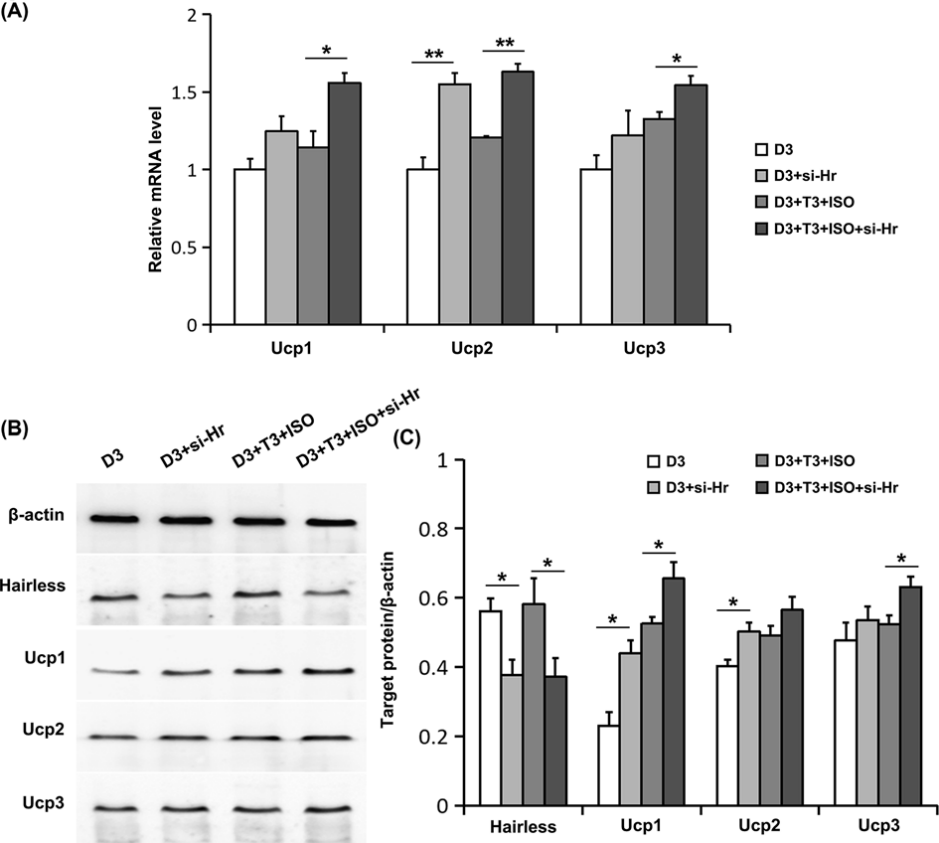
Effects of Hr interference by siRNA on the expression of Ucps regulated by 1,25(OH)_2_D_3_ (**A**) The mRNA levels of Ucps were determined using real-time RT-PCR. (**B**) The protein levels of Hairless and Ucps were measured using Western blot. Blots reflect representative data. (**C**). The level of target protein/β-actin was quantified. Data were expressed as means ± SEM (*n*=3). *P<0.05, **P<0.01.

Then we observed the effects of Hr interference by siRNA on Ucps expression regulated by 1,25(OH)_2_D_3_. Hr interference by siRNA significantly decreased the level of Hairless protein (Figure 5B,C). At the basal condition, there was a slight upstream trend on Ucp1 and Ucp3 mRNA levels in the Hr interference group, although no significant difference was observed (Figure 5A). Importantly, under activated conditions induced by ISO and T3, Ucps mRNA levels were notably elevated in the Hr interference group (Figure 5A). Moreover, the results of Western blot showed the roughly identical effects of Hr interference on Ucps protein expression to the mRNA expression (Figure 5B). The above findings indicate that Hr interference by siRNA alleviates the inhibitory effect of 1,25(OH)_2_D_3_ on Ucps expression.

**Figure 6:**
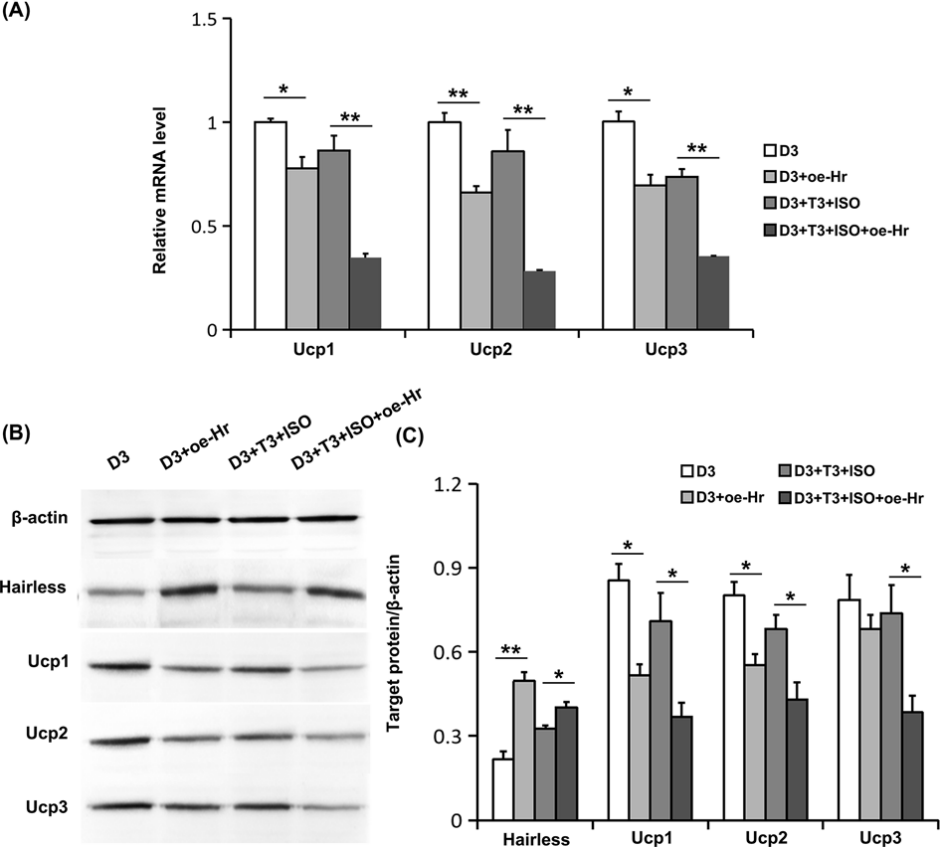
Effects of Hr overexpression on the expression of Ucps regulated by 1,25(OH)_2_D_3_ (**A**) The mRNA levels of Ucps were determined using real-time RT-PCR. (**B**) The protein levels of Hairless and Ucps were measured using Western blot. Blots reflect representative data. (**C**). The level of target protein/β-actin was quantified. Data were expressed as means ± SEM (*n*=3). *P<0.05, **P<0.01.

We further investigated the effect of Hr overexpression on Ucps levels by 1,25(OH)_2_D_3_. It indicated that Hr overexpression further strengthened the down-regulation effects of Ucps by 1,25(OH)_2_D_3_, both in mRNA (Figure 6A) and protein levels (Figure 6B,C).

**Figure 7:**
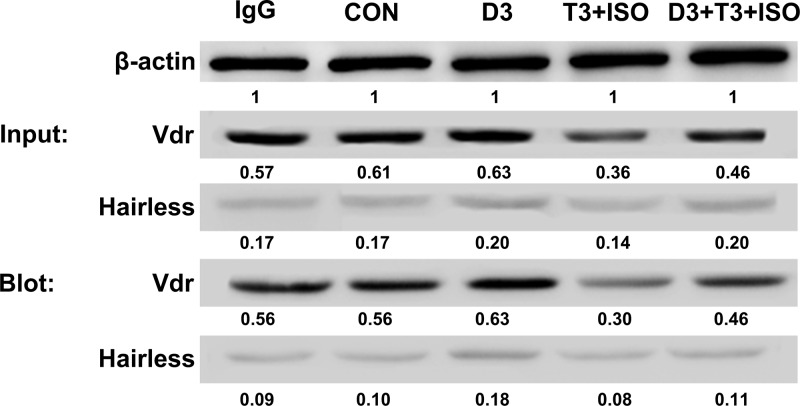
Immunoprecipitation of Vdr and Hairless protein To test for the interaction of Vdr and Hairless in brown adipocyte treated with 1,25(OH)_2_D_3_, immunoprecipitation with anti-Vdr antibody was carried out. Immunoprecipitation (IP) and immunoblot (IB) detection after pull-down by antibodies against Vdr and Hairless in brown adipocyte.

To further determine the interaction of Vdr and Hairless, lysates from the above-mentioned experiment were immunoprecipitated with anti-Vdr antibody. Protein blots of IP fractions were probed for Vdr and reprobed for Hairless. Immunoprecipitation results (Figure 7) revealed that Vdr could interact with Hairless, which might contribute to the decrease in Vdr target gene Ucps expression.

## Discussion

Previous studies have observed that Vdr knockout mice and mice fed with vitamin D deficiency diet are both resistant to high-fat-diet-induced obesity [8,11,13]. One suggested mechanism for this role is that Ucps, central regulators of uncoupled fatty acid oxidation that enhances energy metabolism, increase in WAT and BAT [8,11,13]. Because these studies are *in vivo* experiments, it is unclear whether the phenotype results from a tissue-specific or cell-specific effect. And, the molecular mechanism involved in it remains unresolved. In the present study, we used primary brown adipocytes from neonatal rats to reveal the regulation of Ucps by vitamin D–Vdr system. Not surprisingly, we discovered that 1,25(OH)_2_D_3_ appears to directly down-regulate Ucps expression in brown adipocyte in a Vdr-dependent manner. The result is consistent with the study of Wong et al. [13]. And, we found that the effects of 1,25(OH)_2_D_3_ on Ucps expression are similar whether ISO and T3 exist or not. Previously, Wong et al. observed high energy expenditure and elevated Ucps expression of BAT in Vdr^−/−^ mice [13]. However, Adrβ3 expression was unchanged in the BAT of Vdr^−/−^ mice, and circulating thyroid hormones did not increase compared with WT mice [13]. The above findings suggest that vitamin D regulation of Ucps was not via Adrβ3, and the thyroid hormone was not involved in the regulation of Ucps expression either. That is to say, the regulation of Ucps expression by the VD–Vdr system may well be an independent mechanism.

In contrast with the well-established role of Ucp1 in thermogenesis, the physiological function of Ucp2 and Ucp3 in BAT is still unclear. Ucp2 is widely expressed in most tissues including BAT, while Ucp3 is selectively expressed in skeletal muscle and BAT. Quantification using recombinant Ucp3 revealed that the Ucp3 amount in BAT was nearly one order of magnitude higher than that in muscles and heart [15]. Despite the structural difference between Ucp2 or Ucp3 and Ucp1, there is consensus that Ucp isoforms possess uncoupling functions [16]. In humans, genetic variations of Ucp2 and Ucp3 have been associated with abdominal obesity, type 2 diabetes, and increased serum lipid levels [17,18]. These data suggest Ucp2 and Ucp3 as promising pharmacological targets. Our research showed that VD–Vdr also down-regulated the expression of Ucp2 and Ucp3, as well as Ucp1. It indicated that the VD–Vdr system inhibited strongly uncoupling functions of brown adipocyte. But the true functions of Ucp2 and Ucp3 in BAT deserve further investigation.

The Hairless protein has long been suspected to regulate a stem cell-mediated process, hair cycling, as mutations in the Hr gene cause hair loss in both mice and men [19,20]. Work over the past 20 years has shown clearly that Hairless protein functions as a corepressor for multiple nuclear receptors, including Vdr [21]. Vdr has also been shown to bind Vdr coregulators, which do not directly interact with DNA but are associated with the regulation of gene expression by their modulation of the DNA superstructure [22]. Vdr can recruit and bind nuclear receptor corepressor Hairless to suppresses transcription, which is critical for regulating hair cycling [23]. A previous study that using human patient samples from individuals with hereditary vitamin D-resistant rickets showed that Ucp1 expression is negatively regulated by the Vdr in human cells, but the co-activator interaction is probably not required for the inhibitory activity [12]. Whether or not does Hairless protein, as the usual corepressor, play a role in the down-regulation of Ucps by vitamin D–Vdr system in brown adipocyte? We demonstrated that Hairless protein did exist in rat primary brown adipocyte. And Hairless protein exercised inhibiting effect in the regulation of Ucps expression by vitamin D through binding Vdr. The above findings further elucidate the mechanism of regulation of Ucps expression by vitamin D, which was the innovation of the present study. However, what is on earth the effect of Hairless in human brown adipocyte, what happens next to the binding Hairless protein and Vdr? These aspects also deserve further investigation.

Low serum 25(OH)D is positively associated with obesity or body mass index in adults and children [3]. An adequate vitamin D concentration is essential for growth, development, and health. The Endocrine Society recommends obese children and adults a dose that is two- to three-times higher than for normal-weight ones to satisfy their body’s vitamin D requirement, however, there are no studies that justify this [24,25]. Since obesity induces decreased serum vitamin D level and Vdr signal in adipose tissue, we hypothesize that the latter triggers energy expenditure by increasing expression of Ucps in WAT and BAT, which in turn prevents further development on obesity. That is to say, low vitamin D level and Vdr signal are the adaptation responses to obesity. Then high-dose vitamin D supplement probably has an adverse impact on energy balance. However, an intervention study was used with a treatment of 2000 IU cholecalciferol/day for 7 days. The result shows that this intervention does not affect energy expenditure or substrate metabolism nor gene expression of proteins related to fat metabolism, despite a significant increase in serum 1,25(OH)_2_D_3_ concentration [26]. Nevertheless, the trials had a relatively small sample size and short-duration intervention, and the subjects are only normal-weight young men. Therefore, future well-designed randomized controlled trials are necessary to evaluate the role of vitamin D supplementation on energy balance, especially for obese. It is very important to direct the therapeutic regimen of obese with vitamin D deficiency. The present study shows that vitamin D inhibits the expression of Ucps in brown adipocytes, so vitamin D supplementation for obese children should be cautious, because increased vitamin D levels may inhibit the thermogenesis of brown fat through increasing Vdr expression, which is not beneficial to obesity treatment. The present study reveals the mechanism of Hairless protein in the inhibition of Ucps expression by vitamin D, which may provide a new target for the intervention of obesity.

## Conclusion

In conclusion, our findings in the present study suggest that vitamin D suppresses expression of Ucps in brown adipocyte in a Vdr-dependent manner and the corepressor Hairless protein probably plays a role in the down-regulation.

## Abbreviations

Adrβ3: beta3-adrenoceptor
BAT: brown adipose tissue
DPBS: Dulbecco's phosphate-buffered saline
ECL: enhanced chemiluminescence
Hr: hairless
ISO: isoproterenol
siRNA: small interfering RNA
T3: triiodothyronine
Ucp: uncoupling protein
Ucps: Ucp1–3
VD: vitamin D
Vdr: vitamin D receptor
WAT: white adipose tissue
1,25(OH)_2_D_3_: 1,25-dihydroxyvitamin D3
